# Pulmonary Infection Related to Mimivirus in Patient with Primary Ciliary Dyskinesia

**DOI:** 10.3201/eid2610.191613

**Published:** 2020-10

**Authors:** Fatemeh Sakhaee, Farzam Vaziri, Golnaz Bahramali, Seyed Davar Siadat, Abolfazl Fateh

**Affiliations:** Pasteur Institute of Iran, Tehran, Iran

**Keywords:** pulmonary infection, mimivirus, primary ciliary dyskinesia, viruses, pneumonia, respiratory infections, Iran

## Abstract

Primary ciliary dyskinesia is a rare autosomal recessive disorder that causes oto-sino-pulmonary disease. We report a case of pulmonary infection related to mimivirus in a 10-year-old boy with primary ciliary dyskinesia that was identified using molecular techniques. Our findings indicate that the lineage C of mimivirus may cause pneumonia in humans.

In patients with primary ciliary dyskinesia (PCD), several bacterial pathogens are associated with the occurrence of pulmonary disease. However, in these patients, the many possible causative agents of pneumonia, especially viruses, have not been investigated (*1*). Since the detection of antibodies against mimivirus in patients with pneumonia, the potential role of mimivirus as a respiratory pathogen has been suggested (*2*).

In February 2017, a 10-year-old boy had severe pulmonary infection, caused by *Pseudomonas aeruginosa*. He had been mechanically ventilated for »10 days in the intensive care unit (ICU). One year later, he reported cough, fever, and night perspiration with excessive sputum. His physician speculated the recurrence of *P. aeruginosa* infection and treated him with colistin and ciprofloxacin for 3 weeks. However, symptoms of atypical pneumonia continued after 1 month. 

In May 2018, care was sought agin for the child, who had excessive sputum production, weakness, chills, cough, fever, and night perspiration. He was referred to the Pasteur Institute of Iran for evaluation for nontuberculous mycobacteria. His sputum production, cough, and fever had persisted for 4 months.

The patient’s biological parameters showed elevated leukocyte count (11.9 × 1,000 cells/mL^3^), erythrocyte sedimentation rate (79 mm/h), and C-reactive protein (42.8 mg/L). Computed tomography scan indicated consolidation in the right lower lobe and bilateral basilar infiltrates.

Three sputum and 3 bronchoalveolar lavage (BAL) samples were sent to the laboratory for evaluation for nontuberculous mycobacteria. The results of smear, culture, and PCR for acid-fast bacilli were negative in all samples. We also evaluated what Wijers et al. found to be the most common infectious agents in PCD patients (*3*) but did not detect them in culture or PCR.

We used real-time PCR to identify mimivirus DNA, as previously described (*4*). Five (3 BAL and 2 sputum) of 6 samples were positive for mimivirus. All control samples were negative. We also sequenced the mimivirus genome using an Illumina Hiseq 2000 system (Illumina, https://www.illumina.com). Analysis of a partial genome sequence (»730 kbp) showed 99% homology to megavirus LBA111 (mimivirus lineage C) and the species *Megavirus chilensis*. We named the virus mimivirus PCD-1 (Appendix Figure).

To elucidate the evolutionary correlation between the mimivirus PCD-1 and other mimiviruses, we conducted phylogenetic analyses of 4 genes: the major capsid protein, the VV A18 helicase, the family B-DNA polymerase, and the D5 helicase (Figure). The phylogenetic trees indicated the close correlation of mimivirus PCD-1 with megavirus LBA111.

**Figure:**
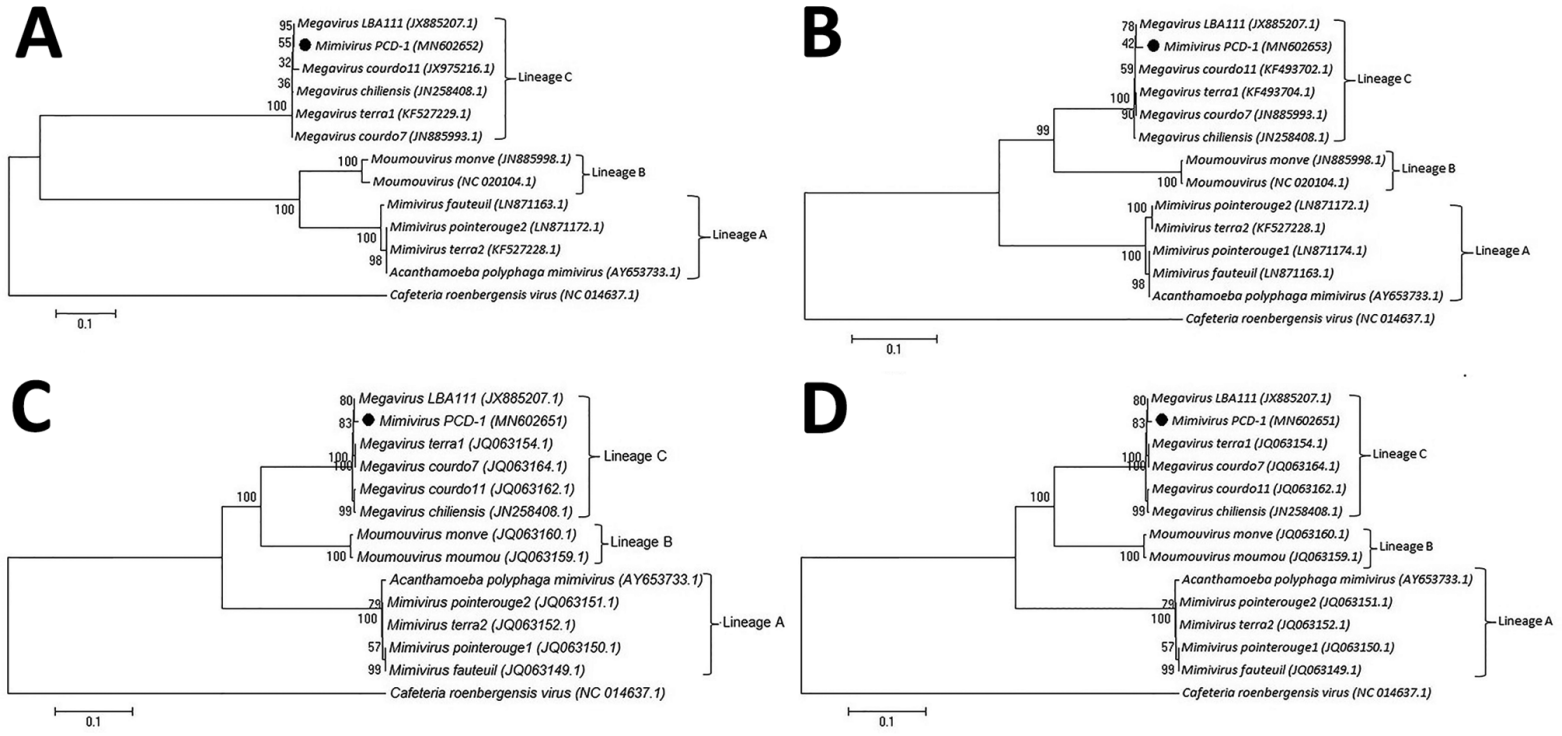
Neighbor-joining tree based on nucleotide acid sequences of mimivirus from a patient in Tehran, Iran (black circles), and reference sequences. A) The major capsid protein. B) The VV A18 helicase. C) The family B DNA polymerase. D) The D5-ATPase-helicase genes. Numbers indicate bootstrap values. Scale bar indicates substitutions per nucleotide position.

The role of giant viruses in human infections remains controversial. Nevertheless, small-scale reports have supported their role (*2*,*4*). We detected mimivirus DNA in sputum and BAL specimens from a 10-year-old boy with PCD in whom pneumonia developed. The negative results of culture and PCR for other pathogens strongly suggest that he had mimivirus pneumonia.

Isolation of mimivirus DNA in respiratory specimens of patients with nosocomial pneumonia who are admitted to ICUs verifies that this virus has reached the respiratory tract in these patients (*5*,*6*). The patient in this report was hospitalized in an ICU for 15 days and was mechanically ventilated for »10 days. Several studies supported the hypothesis that mimivirus occurs in pneumonia patients with ICU ventilation and is probably responsible for pneumonia and should be treated as a class 2 pathogen (*2*,*7*–*9*).

Although 2 previous studies have shown mimivirus infection in lower respiratory BAL specimens (*2*,*4*), we detected the virus in both upper and lower respiratory tracts, including in sputum specimens. The presence of this virus in the upper respiratory tract needs to be considered.

The first mimivirus isolated from respiratory samples belonged to lineage C (*4*). Consistent with this finding, the mimivirus PCD-1 from this patient was also from lineage C. In addition, another lineage C mimivirus (Shan virus) was found from the feces of a patient with pneumonia in Tunisia (*10*). This lineage of mimivirus may be responsible for pulmonary infection in patients; however, future research needs to confirm this result.

In summary, we detected mimivirus in a patient with primary ciliary dyskinesia who had pneumonia develop. Whether mimivirus is a causative agent of pneumonia or only extremely immunogenic is unclear, but clinicians should be aware of the potential role of this virus in human infections.

Appendix Additional information for study of pulmonary infection related to mimivirus in patient with primary ciliary dyskinesia, Iran.
