# Supplementary material for: Pulmonary Infection Related to Mimivirus in Patient with Primary Ciliary Dyskinesia
Source: Emerg Infect Dis. 2020 Oct;26(10):2524–6. doi: 10.3201/eid2610.191613 (PMC7510730; doi:10.3201/eid2610.191613)
Supplement: Appendix — Additional information for study of pulmonary infection related to mimivirus in patient with primary ciliary dyskinesia, Iran. [file 19-1613-Techapp-s1.pdf]

Article DOI: https://doi.org/10.3201/eid2610.191613

Pulmonary Infection Related to Mimivirus in Patient with Primary Ciliary Dyskinesia

Appendix

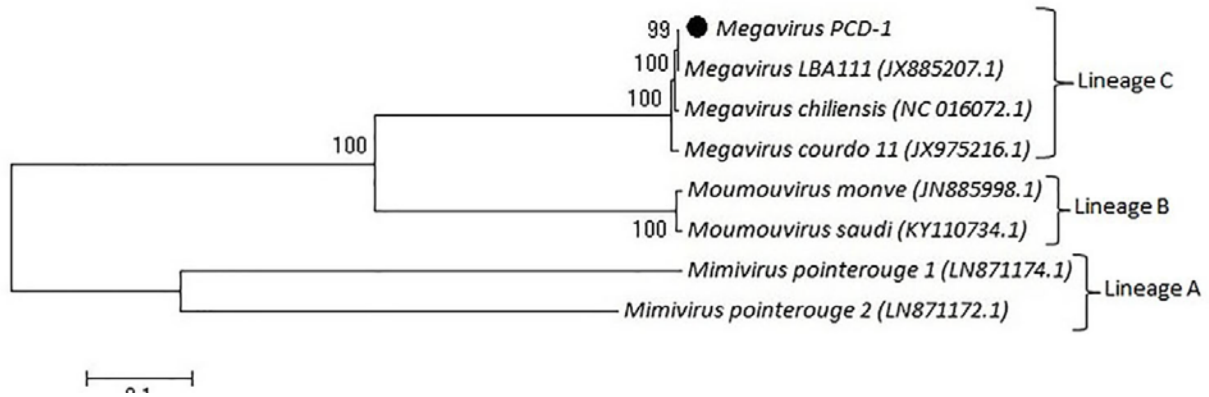

Appendix Figure. Whole-genome phylogenetic tree of Mimiviridae. Scale bar indicates substitutions per nucleotide position.
